# Selective delivery of curcumin to breast cancer cells by self-targeting apoferritin nanocages with pH-responsive and low toxicity

**DOI:** 10.1080/10717544.2022.2056662

**Published:** 2022-04-01

**Authors:** Peng Ji, Xianglong Wang, Jiabing Yin, Yi Mou, Haiqin Huang, Zhenkun Ren

**Affiliations:** a College of Pharmacy and Chemistry & Chemical Engineering, Jiangsu Provincial Key Laboratory of Chiral Pharmaceutical Chemicals Biologically Manufacturing, Taizhou University, Taizhou, PR China; b School of Pharmacy, Nantong University, Nantong, PR China; c The Third Hospital Affiliated of Jinzhou Medical University, Jinzhou, PR China

**Keywords:** Human heavy chain apoferritin drug carrier (HFn), curcumin, anti-tumor, tumor self-targeting, ROS

## Abstract

Breast cancer is prevalent and diverse with significantly high incidence and mortality rates. Curcumin (Cur), a polyphenol component of turmeric, has been widely recognized as having strong anti-breast cancer activity. However, its anti-cancer efficiency is largely impaired by some of its concomitant negative properties, including its poor solubility, low cellular uptake, and severe reported side effects. Hence, the necessity arises to develop a novel low-toxic and high-efficiency targeting drug delivery system (DDS). In this study, we developed a pH-sensitive tumor self-targeting DDS (Cur@HFn) based on self-assembled HFn loaded with Cur, in which Cur was encapsulated into HFn cavity by using a disassembly/reassembly strategy, and the Cur@HFn was characterized by ultraviolet–visible (UV–vis), dynamic light scattering (DLS), and transmission electron microscope (TEM). A variety of breast cancer cell models were built to evaluate cytotoxicity, apoptosis, targeting properties, and uptake mechanism of the Cur@HFn. The pharmacodynamics was also evaluated in tumor (4T1) bearing mice after intravenous injection. *In vitro* release experiments showed that Cur@HFn is pH sensitive and shows sustained drug release under slightly acidic conditions. Compared with Cur, Cur@HFn has stronger cytotoxicity, cellular uptake, and targeting performance. Our study supported that Cur@HFn has a higher *in vivo* therapeutic effect and lower systemic toxicity. The safety evaluation results indicated that Cur@HFn has no hematotoxicity, hepatotoxicity, and nephrotoxicity. The findings of the present study showed that the Cur@HFn has been successfully prepared and has potential application value in the treatment of breast cancer.

## Introduction

Cancer is a leading cause of death in the world (Cheng et al., 2020) and the most common type is breast cancer. The incidence rate of breast cancer among women ranks first in the world, and the mortality rate ranks second. Breast cancer affects approximately 2–2.5 million women worldwide each year (Jain et al., 2020). In 2018, about 627,000 women died of breast cancer. Global cancer statistics suggest that breast cancer is the most commonly diagnosed cancer among females (24.2%) and also the leading cause of cancer death (15%) (Xiao et al., 2021). There are three basic treatment options for breast cancer: surgery, chemotherapy, and radiotherapy. Due to the refractory and metastatic nature of breast cancer, most female patients choose to receive breast cancer surgery first and then rely on adjuvant chemotherapy to reduce the risk of recurrence and to control metastasis (Yue et al., 2020). However, conventional synthetic chemotherapeutics generally suffer from poor water solubility, drug resistance, short blood circulation time, insufficient curative effect, nonspecific distribution, and serious side effects, which greatly hinder their clinical application (Alven & Aderibigbe, 2020). Thus, there is a need to develop more chemotherapy drugs with low-toxic and high efficiency so that these limitations might be overcome. To resolve this issue, the successful application of natural plant medicines (such as artemisinin, developed by Tu Youyou) in recent years has brought a new avenue for researchers. In the past 30 years, nearly 80% of all drugs approved by the FDA for cancer treatment are either natural products per se or derivatives (Mansourizadeh et al., 2020).

Curcumin (Cur), a polyphenol flavonoid, is extracted from the rhizome of Curcuma longa Linn. Cur has a wide range of biological and pharmacological activities, including analgesic, antiseptic, anti-atherosclerosis, antioxidant, and anti-inflammatory activities (Ji et al., 2020a). It is often used in Chinese food recipes and traditional folk medicine prescriptions. In recent years, a plethora of cytotoxicity and animal experiments have shown that Cur has good anti-cancer activity, especially in the treatment of breast cancer, prostate cancer, lung cancer, and ovarian cancer (Ma et al., 2019). Its mechanism is to inhibit P-gp and down-regulate nuclear factor kappa-light-chain-enhancer of activated B cell (NF-κB) and phosphoinositide 3-kinase (PI3K)/protein kinase B (Akt) pathways, inducing apoptosis by suppressing the growth of cancer cells (Kim et al., 2021). Clinical trials have proved that Cur has few side effects and high safety, and the safe dose can be as high as 12 g per day (Kumari et al., 2020). The National Cancer Institute (NCI) has listed it as third-generation cancer chemoprevention and chemotherapy drug. Although Cur has a good prospect in the treatment of breast cancer, its clinical application is subject to many limitations, such as poor water solubility, fast metabolism, and nonspecific biodistribution (Sampath et al., 2020). To address these bottlenecks, researchers have developed a variety of Cur nano-drugs, including polymer micelles, liposomes, solid dispersions, microemulsions, and so on (Mahmoudi et al., 2021). However, traditional nanocarriers often have disadvantages, such as poor biocompatibility, unsatisfactory delivery efficiency, strong toxicity, poor stability, and complex synthesis process. Therefore, it is highly necessary to develop a targeted specific drug delivery system (DDS) with better performance to meet these challenges.

Bio-nanomaterials have attracted more and more attention in the field of drug delivery due to their specific characteristics, such as precisely defined size, biocompatibility, and biodegradability (Ye et al., 2021b). Recombinant human heavy chain apoferritin (HFn), a hollow cage-like molecule composed of 24 protein subunits, has a molecular weight of 450 kDa, an inner diameter of 8 nm, and an outer diameter of 12 nm. Compared with other nanomaterials, HFn has the following unique characteristics (Pandolfi et al., 2017; He et al., 2019; Ji et al., 2019): first, HFn is a natural endogenous substance with good biocompatibility and low immunogenicity; second, HFn has a small and uniform particle size (<30 nm), enabling it to cross biological barriers and achieve deep penetration; third, it has pH-sensitive self-assembly properties. At strong acidic or alkaline pH, the shell of HFn is unfolded into a single subunit. When the pH is neutral, the shell is folded into the original quaternary structure; fourth, HFn has self-targeting properties. HFn is specifically recognized by transferrin receptor 1 (TfR1), which is overexpressed in several human breast cancers including 4T1 and MDA-MB-231, and promotes the intracellularization of these nanoparticles. These properties together make HFn an attractive new drug delivery carrier for cancer treatment.

**Scheme 1.**
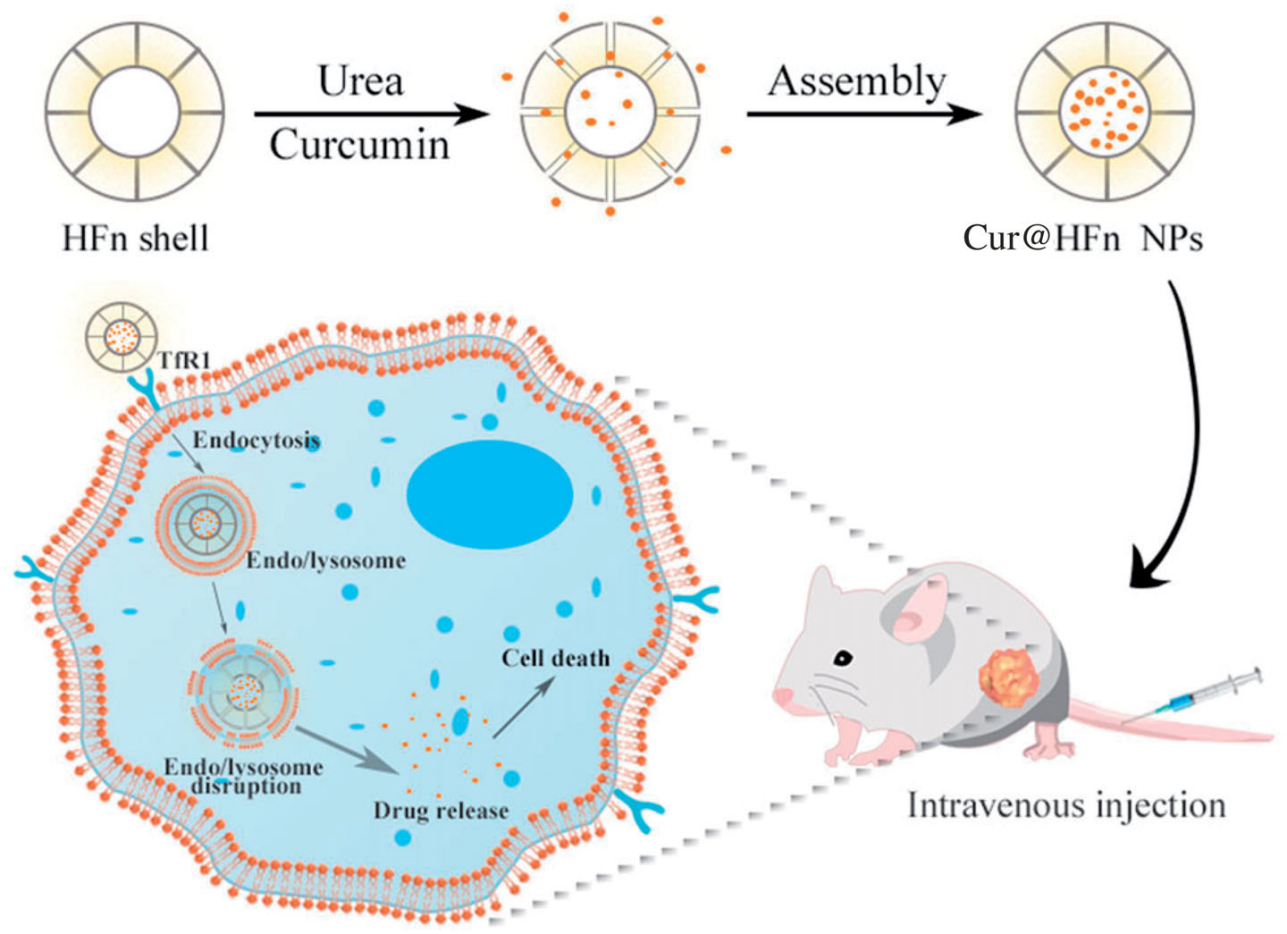
Schematic illustration of Cur@HFn for anticancer therapy.

In this work, we designed and proposed a strategy based on HFn nanocages as the delivery carrier of natural plant drugs, which can give full play to the advantages of HFn as endogenous substances with high biocompatibility and high specificity for active targeting. The method of disassembly/assembly is used to load Cur into the HFn cavity to prepare pH-sensitive targeted therapeutic nano-drugs (Cur@HFn), in an attempt to study the effect of Cur in the treatment of breast cancer (Scheme 1). The morphology, zeta potential, and size of Cur@HFn were characterized by dynamic light scattering (DLS) and transmission electron microscope (TEM). An ultraviolet–visible (UV–vis) spectrophotometer was utilized to measure and record the controlled release of Cur at different pH values. The murine breast cancer cell line (4T1) and human breast cancer cell line (MDA-MB-231) were cultured *in vitro* to study the effects of Cur and Cur@HFn on cytotoxicity, apoptosis, reactive oxygen species (ROS) production, and cellular uptake. Finally, the 4T1 tumor-bearing mouse model was designed to verify the *in vivo* therapeutic effect on breast cancer. The purpose of this study is: (i) to formulate and characterize Cur@HFn and (ii) to evaluate the anti-cancer effect of Cur@HFn in breast cancer cells and tumor-bearing models.

## Materials and methods

### Materials

Cur (Mw = 368.38, purity ≥98%), sodium hydroxide, and trisodium citrate were purchased from Macklin Biochemical Co., Ltd. (Shanghai, China). The 3-(4,5-dimethylthiazol-2-yl)-2,5-diphenyltetrazolium bromide (MTT) was provided by Sigma-Aldrich Co. (St. Louis, MO). The dialysis bags were obtained from Sinopharm Chemical Reagent Co. Ltd. (Shanghai, China). Dulbecco’s modified Eagle medium (DMEM), penicillin–streptomycin, and fetal bovine serum (FBS) was purchased from Gibco BRL (Gaithersburg, MD). ROS detection kit was obtained from Bio Lebo Technology Co., Ltd. (Beijing, China). Hoechst 33342 was purchased from Beyotime Biotechnology (Nanjing, China). The water used in the experiments was deionized. Other reagents were of analytic grade. MDA-MB-231 and 4T1 were obtained from the American Type Culture Collection (ATCC) (Manassas, VA). Female Balb/c mice (4–5 weeks old) were obtained from the Laboratory Animal Center of Nantong University.

### Preparation of Cur@HFn

HFn was obtained through *E. coli* expression according to our previous report (Ji et al., 2019). The encapsulation of Cur into holes of HFn was done by using a disassembly/reassembly strategy (Liang et al., 2014). The procedures can be briefly stated in the following. HFn was dissolved in an 8 M urea solution to obtain the final protein concentration (4 mg/mL). After HFn was fully dissociated, 0.1 M sodium hydroxide Cur solution (20 mg/mL) was added. The mixture was incubated at 60 °C for 1 h and then transferred to a dialysis bag (MWCO = 3500 Da), followed by dialyzing with gradient urea buffer (7, 5, 3, 2, 1 M, 4 h each). Finally, the obtained Cur@HFn solution was dialyzed (MWCO = 8000–14,000 Da) with phosphate buffer (pH 7.4, 0.025 M) to remove unencapsulated drugs.

### Characterization of Cur@HFn

The average particle size (*z*-average), polydispersity index (PDI), and zeta potential (*ζ*) of the Cur@HFn were measured by DLS technique using a Zetasizer Nano ZS^®^ (Malvern Instruments, Malvern, UK). Morphology of the as-formed Cur@HFn was observed and imaged by TEM (alpha300R, WI Tec, Ulm, Germany). The Cur content of the drug was determined by the UV–vis at *λ*_max_=430 nm using the calibration curve. Cur molecules were released by adjusting the pH value of the Cur@HFn sample (500 μL) to 2.5 with 1 M HCl. Drug loading capacity (%, DL) was calculated using the following equations: drug loading capacity (%)=weight of encapsulated Cur/weight of Cur@HFn × 100%. To evaluate the short-term stability of Cur@HFn, an appropriate amount of Cur@HFn and Cur (dissolved in DMSO at 50 mM) were dispersed in PBS (pH 7.4) containing 10% FBS. The test was conducted at 4 °C, 25 °C, and 37 °C for 5 d. The absorbance of Cur was measured regularly every day, and the content of Cur was calculated. Each measurement was performed in triplicate. The surface chemical structure of the samples was examined by Fourier infrared (FTIR) spectroscopy. The sample of 2 mg Cur@HFn was weighed, ground well together with 300 mg potassium bromide, dried and pressed, and scanned in the wavenumber range of 4000–400 cm^−1^.

To verify whether Cur@HFn can release drugs in response to the slightly acidic environment of the tumor, PBS buffer (0.1 M) with different pH (pH 8.0, pH 7.4, pH 6.0, and pH 5.0) was used as the release medium (Liu et al., 2020). The free Cur or Cur@HFn (containing 1000 μg Cur) was put into a dialysis bag (MWCO = 8000–14,000 Da), after which the bag was immersed in a 40 mL dissolving medium. The dialysis bag was then kept in 37 °C constant temperature water, stirred gently (shaking rate of 100 rpm). Samples were taken at predetermined time intervals, and the absorbance of the released Cur in the dissolution medium was determined by a UV–vis. Cumulative release rate (%)= [*c_n_*×*V*+$\sum_{i=1}^{n-1}c_{i}$×*V*_i_]/*W*_Cur_×100%, where *W*_Cur_ denotes the total amount of Cur in the dialysis bag; *V*_i_ denotes the volume of dialysate aspirated each time; *V* denotes the total volume of buffer; *c*_i_ and *c_n_* denote the concentration of Cur, where *i* and *n* are the number of sampling times.

### Cell culture conditions and treatment

MDA-MB-231 and 4T1 cells were cultured in DMEM media containing 10% FBS and penicillin–streptomycin (100 IU/mL to 100 μg/mL) under a humidified atmosphere of 5% CO_2_ 95% air at 37 °C.

### *In vitro* cytotoxicity

To explore the therapeutic effect of Cur@HFn, the anti-tumor effects of Cur and Cur@HFn on cultured cells were evaluated by the MTT experiment (Chen et al., 2020a; Liu et al., 2021; Wei et al., 2022). Two kinds of cancer cell suspensions in the exponential growth phase were seeded in 96-well plates (100 μL medium per well) at a density (5 × 10^4^ cells/well) and incubated for 24 h. Then, the cells were incubated with different concentrations of HFn (0–1000 μg/mL), free Cur, and Cur@HFn (Cur concentrations: 60, 40, 20, 10, and 5 μg/mL) at continuous concentrations for 24 h. After that, 10 μL of MTT solution (5 mg/mL) was added to each well. After 4 h of incubation, formazan was dissolved in DMSO, and a microplate reader (Thermo Fisher Scientific, Waltham, MA) was used to measure the OD (optical density) value at a wavelength of 490 nm. The data were processed using GraphPad Prism 7.0 (La Jolla, CA).

### Cellular uptake

The intrinsic fluorescence spectrum of Cur can be used to study the cellular uptake behavior of Cur@HFn. 4T1 cells were placed in a six-well plate and incubated overnight to adhere to the wall, and then Cur@HFn (50 μg/mL Cur) was added. After incubating at 37 °C for 2 and 6 h, the medium was discarded. Cells were washed with PBS three times and fixed with 4% p-formaldehyde. Finally, the cells were imaged by fluorescence microscope under 488 nm excitation, and the ImageJ (Bethesda, MD) was employed to quantify the fluorescence intensity of Cur. In addition, the absorption efficiencies of free Cur and Cur@HFn were also compared at 37 °C for 6 h.

### Mechanism of cellular uptake of Cur@HFn

According to previously reported methods (Ji et al., 2020b), different inhibitors, including sodium azide, chlorpromazine, genistein, and amiloride, were used to study the uptake mechanism of Cur@HFn in 4T1 cells by flow cytometry. Before adding drugs, cells were pretreated with endocytic inhibitors for 1 h, including sodium azide (1 mg/mL), chlorpromazine (8.5 μg/mL), genistein (56.75 μg/mL), and amiloride (133 μg/mL) and cultured. Taking the cells pretreated with no inhibitor as the control, the cell uptake rate of the inhibitor-containing treatment group was significantly reduced, indicating the endocytic pathway of Cur@HFn.

### Apoptosis assay and ROS detection assay

The Annexin V-FITC apoptosis detection kit was adopted to analyze cell apoptosis (Wang et al., 2021). In short, a six-well plate (5 × 10^4^ cells/well) was used to culture 4T1 cells. At 80% confluency, the cells were incubated with free Cur and Cur@HFn (Cur concentration: 50 μg/mL) for 24 h. All cells were collected and stained with 5 μL Annexin-V-FITC and 5 μL propidium iodide (PI). Subsequently, the fluorescence intensity was measured by flow cytometry and analyzed using FlowJo software (Lin et al., 2018). In addition, the cells were rinsed with PBS and fixed, followed by the staining of the nucleus by DAPI (10 μg/mL), and cells were imaged by CLSM. The special morphological feature of apoptotic cell was observed with electron microscope.

A ROS detection kit was used to measure intracellular ROS. In short, 4T1 cells were seeded in a six-well plate at a cell density of 5 × 10^4^/well and adhered. The cells were then incubated with Cur and Cur@HFn (Cur concentration: 50 μg/mL) for 6 h. Discard the medium, wash and stain with 2′,7′-dichlorofluorescein diacetate at 37 °C for 20 min. The cells were washed with PBS three times, fixed with 4% paraformaldehyde, and observed under a fluorescence microscope at the excitation wavelength of 488 nm.

### Therapeutic study

4T1 tumor-bearing mice were used to evaluate the anti-tumor efficacy of Cur@HFn *in vivo* (Kim et al., 2021; Yu et al., 2021). Female mice were inoculated with a suspension of 4T1 cells in physiological saline (100 μL, 1 × 10^6^ cells) into the right mammary gland. When the tumor volume reached 100 mm^3^, the mice were randomly divided into three groups (*n* = 5). Normal saline, Cur, and Cur@HFn (Cur dose 20 mg/kg) were injected through the tail vein every two days for a total of six times. The survival rate was monitored from the second day after treatment. The mice were sacrificed on the 13th day, the main organs and tumors were removed, and histological analysis (H&E staining) was performed. A trained pathologist performed blinded histological analysis of the tissues at the UNMC core facility.

### *In vivo* safety evaluation

The healthy Balb/c mice were randomly divided into two groups. Normal saline and Cur@HFn (20 mg/kg Cur of body weight) were injected via the tail vein every two days for 12 days. Blood samples were collected from the eyeballs of mice on day 13, and hematology and blood biochemical analysis were performed (Kuang et al., 2021).

### Statistical analysis

GraphPad Prism 7.0 statistical software (La Jolla, CA) was used for data analysis, and one-way analysis of variance (one-way ANOVA) was used for comparison among multiple groups. Data are expressed as mean ± standard deviation, *p* < .05 as well as *p* < .01 means the difference is statistically significant.

## Results and discussion

### Preparation and characterization of Cur@HFn

**Figure 1.**
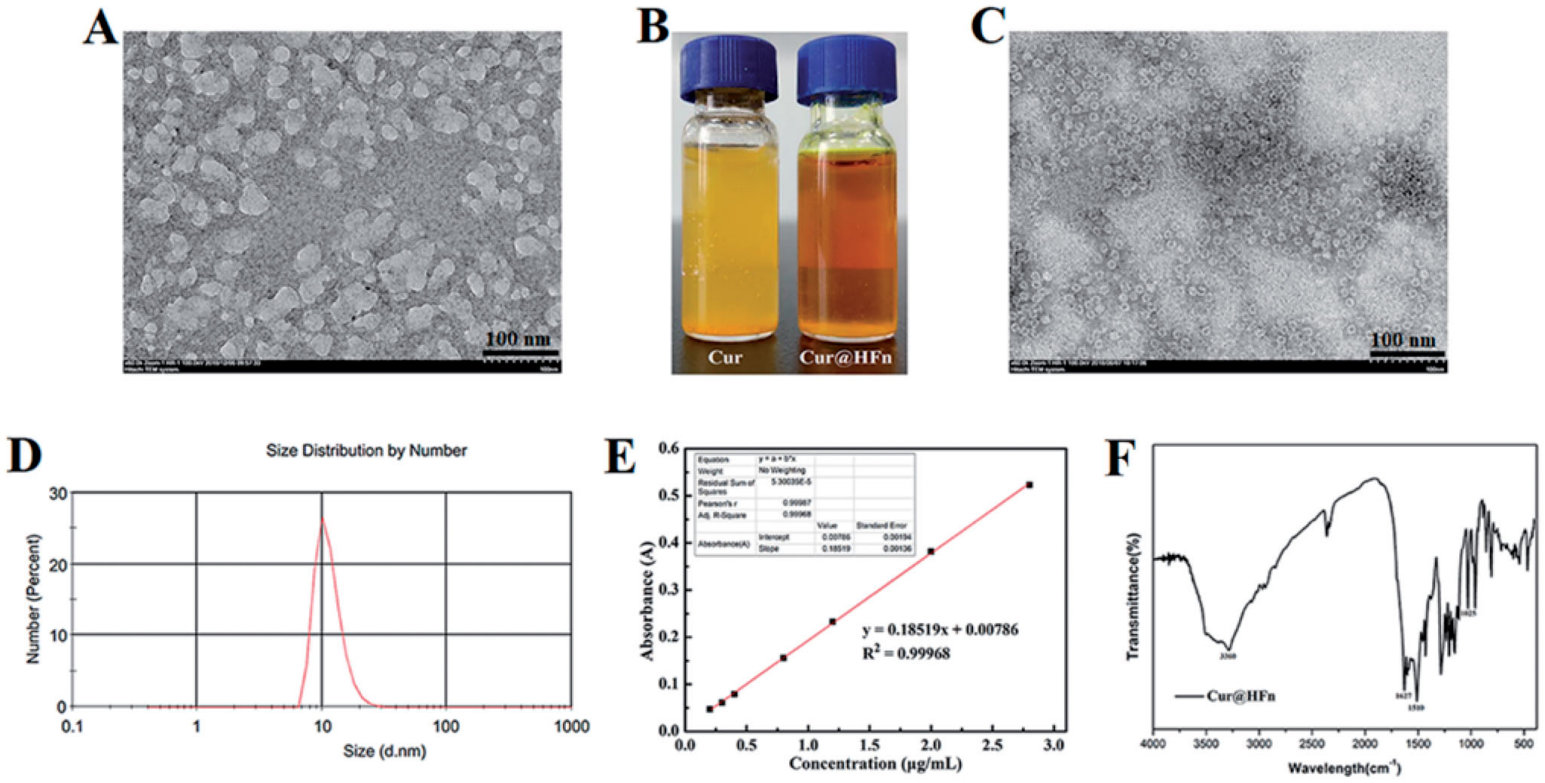
Characterization of the Cur@HFn nanocage. (A) TEM image of disassembled HFn with urea. (B) The appearance of Cur and Cur@HFn. (C) TEM image of Cur@HFn. (D) DLS of Cur@HFn. (E) The quantification of Cur by UV–vis. (F) Infrared spectra of Cur@HFn.

HFn decomposes into its subunits in an acidic pH environment and reassembles after returning to a neutral pH, which is one of the most commonly used drug loading methods (Kim et al., 2011). However, recent studies have found that treatment with acidic pH will irreversibly destroy HFn and form holes on the surface of the nanocage (Inoue et al., 2021). Therefore, we chose mild urea treatment, reassembled after gradual removal of urea, and encapsulated the Cur molecules in HFn nanocages. As shown in Figure 1(A), after treatment with 8 M urea, HFn expands and opens the hydrophobic channel to allow the hydrophobic Cur to enter the cavity and bind through non-covalent bonds. After gradually removing the urea and reassembling it to obtain Cur@HFn, the appearance of Cur@HFn is clear and transparent, and the water solubility of Cur is improved (Figure 1(B)). TEM results showed that the morphology of Cur@HFn is a spherical cage structure with uniform distribution and a diameter of about 13 nm (Figure 1(C)). DLS analysis showed that the hydrodynamic diameter of Cur@HFn was 19.6 nm with a PDI of 0.272 (Figure 1(D)), which was consistent with the results of TEM analysis. The DLS measurement also revealed that the zeta potential of Cur@HFn was −10.8 mV, indicating good biocompatibility. The findings indicate that the loading of Cur will not affect the structural conformation and monodispersed state of HFn, and the Cur@HFn delivery system has good physical stability and uniform distribution. In addition, the slight negative charge on the surface of Cur@HFn and the appropriate particle size can also help shield the reticuloendothelial system, prolong blood circulation and enhance cell uptake in the body (Yu et al., 2021). According to Figure 1(E), with absorbance (*y*) as the ordinate and concentration (*x*) as the abscissa, the standard curve equation of Cur is obtained: *y* = 0.18519*x* + 0.00786 (*R*^2^=0.99968). Calculate the DL (5.71%, mg/mg) of Cur@HFn according to the standard curve. The results show that the compatibility between Cur and protein is high, and the drug loading ability of HFn nanocages is excellent, which can meet the needs of follow-up research. The infrared spectrum of Cur@HFn is shown in Figure 1(F). The Cur molecular structure is mainly composed of a benzene ring, –OH, C=O, –OCH_3_, etc. The characteristic absorption peaks of Cur are still clearly visible in the infrared spectrum curve, which can be attributed to the phenolic hydroxyl stretching vibration at 3360 cm^−1^, the stretching vibration of C=O double bond at 1627 cm^−1^, the benzene ring structure near 1510 cm^−1^, and the C–O–C vibration peak at 1025 cm^−1^. No new characteristic peaks were found, indicating that the HFn carrier was successfully loaded with Cur, there was only physical adsorption between Cur and the carrier material, and no new material was generated.

### *In vitro* stability and pH-controlled release

**Figure 2.**
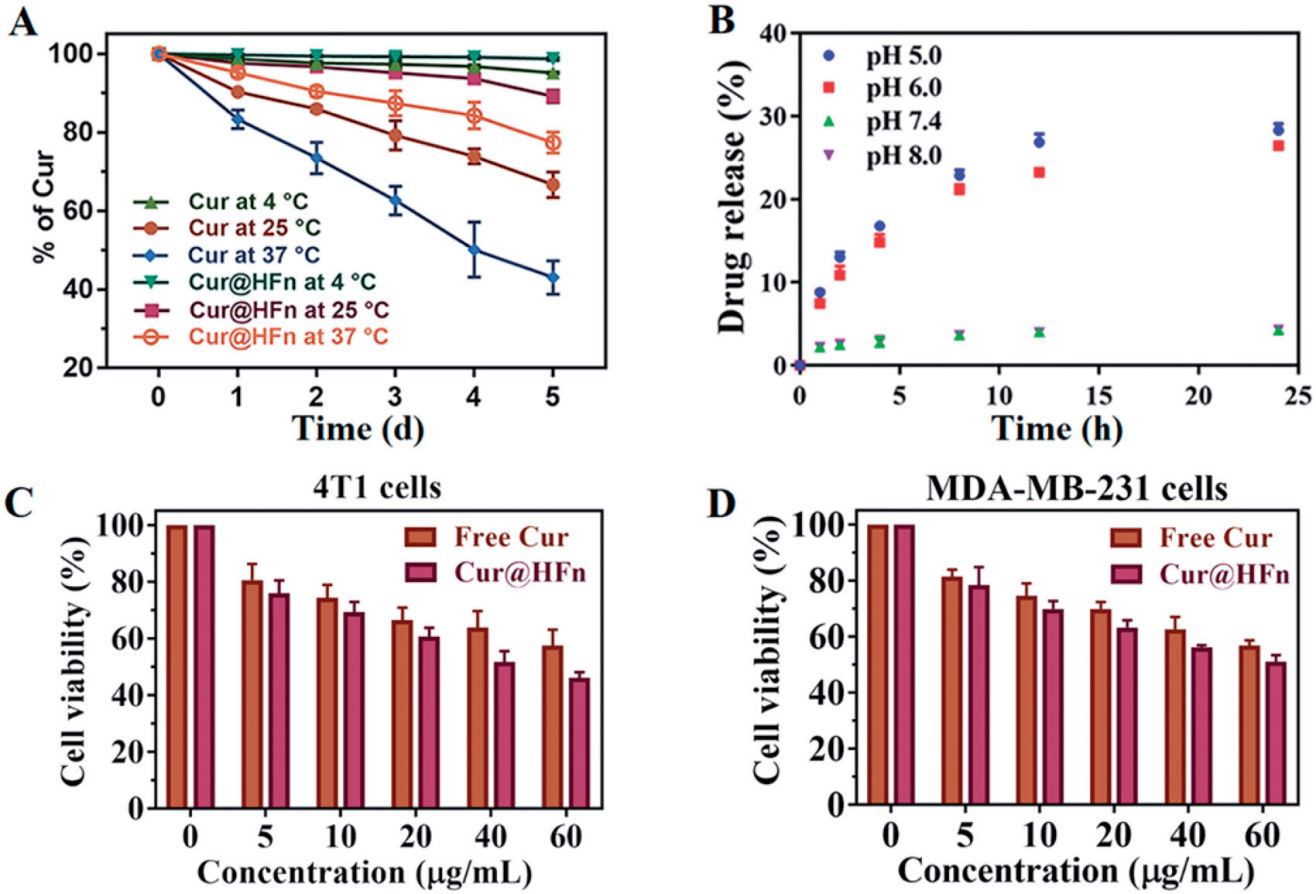
(A) Stability test for Cur and Cur@HFn at 4 °C, 25 °C, and 37 °C. (B) The release of Cur from Cur@HFn nanocages at different pH conditions. (C, D) *In vitro* cytotoxicity evaluation of Cur and Cur@HFn in MDA-MB-231 and 4T1 cells (mean ± SD, *n*= 3).

The stability of nanocarriers under physiological conditions has a greater impact on the efficiency of carrier delivery. Therefore, 10% FBS in PBS that simulates physiological conditions is usually used to predict the stability of nanocarriers *in vivo* (Chen et al., 2017; Lollo et al., 2018). According to Figure 2(A), the Cur content in the free Cur and Cur@HFn groups decreased slowly at 4 °C and both were not less than 95%. However, the degradation of the Cur group was faster at both room temperature and 37 °C, and the degradation of the Cur@HFn group was slower than the free Cur. These results indicated that Cur@HFn is suitable for intravenous administration, can significantly improve the chemical and *in vivo* stability of the molecule, will not affect the delivery efficiency of the carrier, and thus provide a guarantee for efficient treatment *in vivo*. To verify the pH-dependent drug release mechanism of Cur@HFn, pH 7.4 and 5.0 media were used to simulate physiological conditions and lysosomal environment at 37 °C (Liu et al., 2020). At pH 7.4 and pH 8.0, Cur@HFn released a small amount of Cur during 24 h of dialysis (see Figure 2(B)), indicating that Cur@HFn is stable under physiological conditions. However, the release of Cur from HFn nanocages was relatively rapid at pH 6.0 and pH 5.0, reaching 26% and 28% within 24 h, respectively, implying that when Cur@HFn is taken up by cancer cells, its cargo is likely to be released rapidly for efficacy in the slightly acidic environment of the tumor. The results proved that Cur@HFn is a potential acid-responsive DDS.

### *In vitro* cellular toxicity test and antitumor activity

The cytotoxicity of the DDS is one of the vital evaluation factors for the application of nanomaterials *in vivo* (Luan et al., 2019). The cytotoxic effects of HFn, Cur, and Cur@HFn on 4T1 and MDA-MB-231 cells were evaluated by MTT assay. As shown in Figure S1, the cell line treated with nanocarrier HFn (0–1000 μg/mL) for 24 h did not cause any significant toxicity, and the cell viability remained above 85%, which indicates that HFn has good biocompatibility. The MTT test (Figure 2(C,D)) also evaluated the anticancer potential of Cur and Cur@HFn. After both cell lines were incubated with a series of drug concentrations of Cur and Cur@HFn for 24 h, both Cur and Cur@HFn showed concentration-dependent cytotoxicity. Compared with the same concentration of Cur, the higher concentration of Cur@HFn has significantly higher cytotoxicity in breast cancer cells (*p* < .05). On the one hand, this may be due to their different uptake mechanisms. Cur directly enters the cell through molecular diffusion, while the internalization of Cur@HFn is through TfR1 receptor-mediated endocytosis (Ma et al., 2021). On the other hand, because Cur has poor water solubility, Cur@HFn exhibits high water dispersibility and consequently endow Cur with higher bioavailability (Madhusudana Rao et al., 2015).

### Cellular uptake

**Figure 3.**
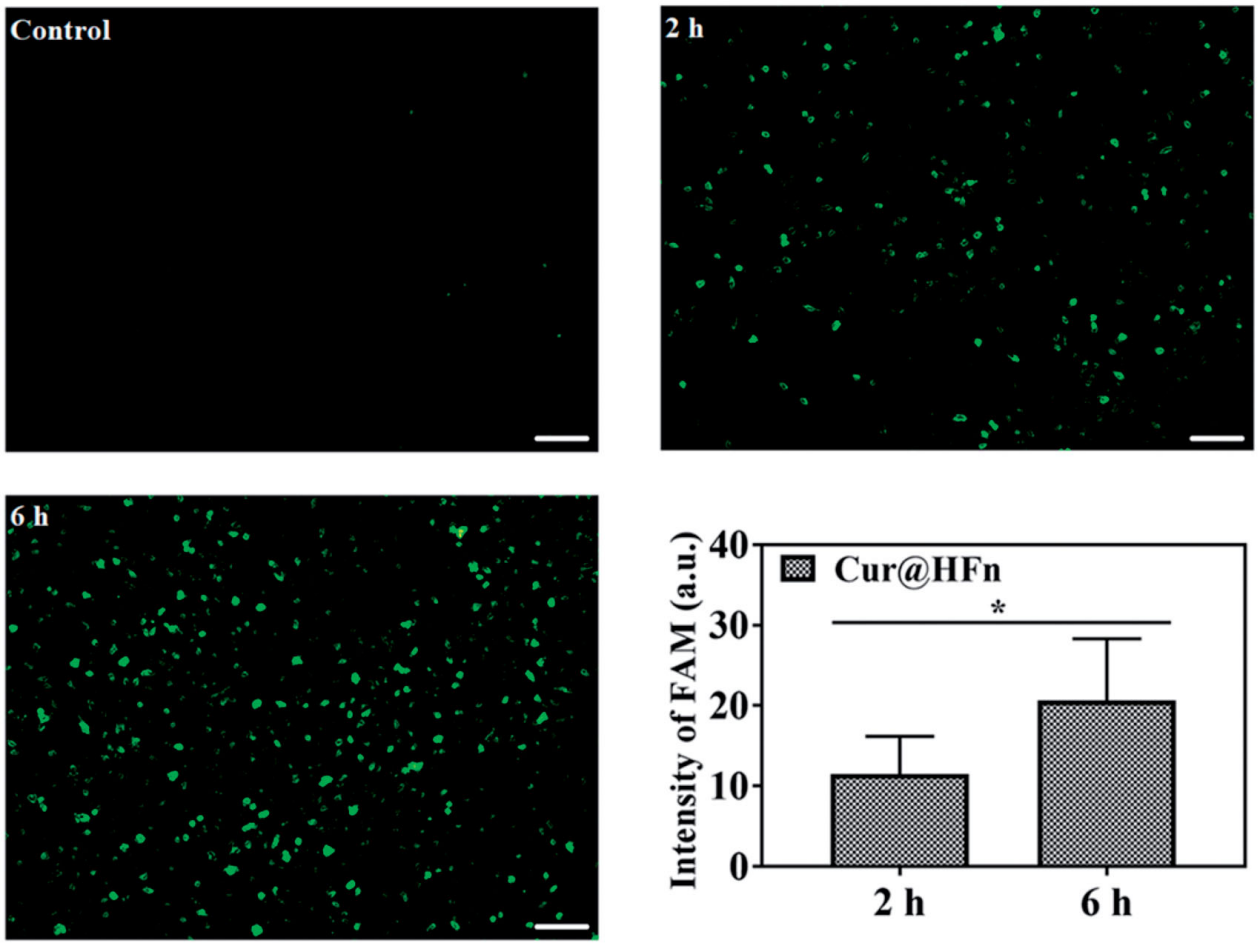
Fluorescence microscopy images of 4T1 cells after being incubated with Cur@HFn for 2 h and 6 h (scale bar: 100 μm). The mean fluorescence intensity of 2 h and 6 h, *p< .05.

Efficient cellular uptake is necessary for the drug to produce its therapeutic efficacy (Yin et al., 2018). The internalization of 4T1 cancer cells treated with Cur and Cur@HFn during different incubation times was observed through a fluorescence microscope (Chen et al., 2020b). The cell uptake of breast cancer cells with or without treatment is shown in Figure 3. The treatment groups at different incubation times all showed green fluorescence, indicating that the cancer cells can effectively absorb Cur@HFn. In addition, the green fluorescence increased from 2 h to 6 h with time and became more intense, revealing the time-dependent behavior of Cur@HFn cell uptake, which is consistent with the above-mentioned MTT results. Figure S2 shows a follow-up observation of the subcellular distribution of Cur and Cur@HFn incubations for 6 h. The fluorescence intensity of the Cur@HFn group was significantly higher than that of the Cur group for the same incubation time (*p*<.05), indicating that the uptake efficiency of Cur@HFn was higher than that of free Cur. The reason is that Cur@HFn has good cellular uptake ability due to the existence of TfR1 receptor-mediated uptake.

### Internalization mechanism

**Figure 4.**
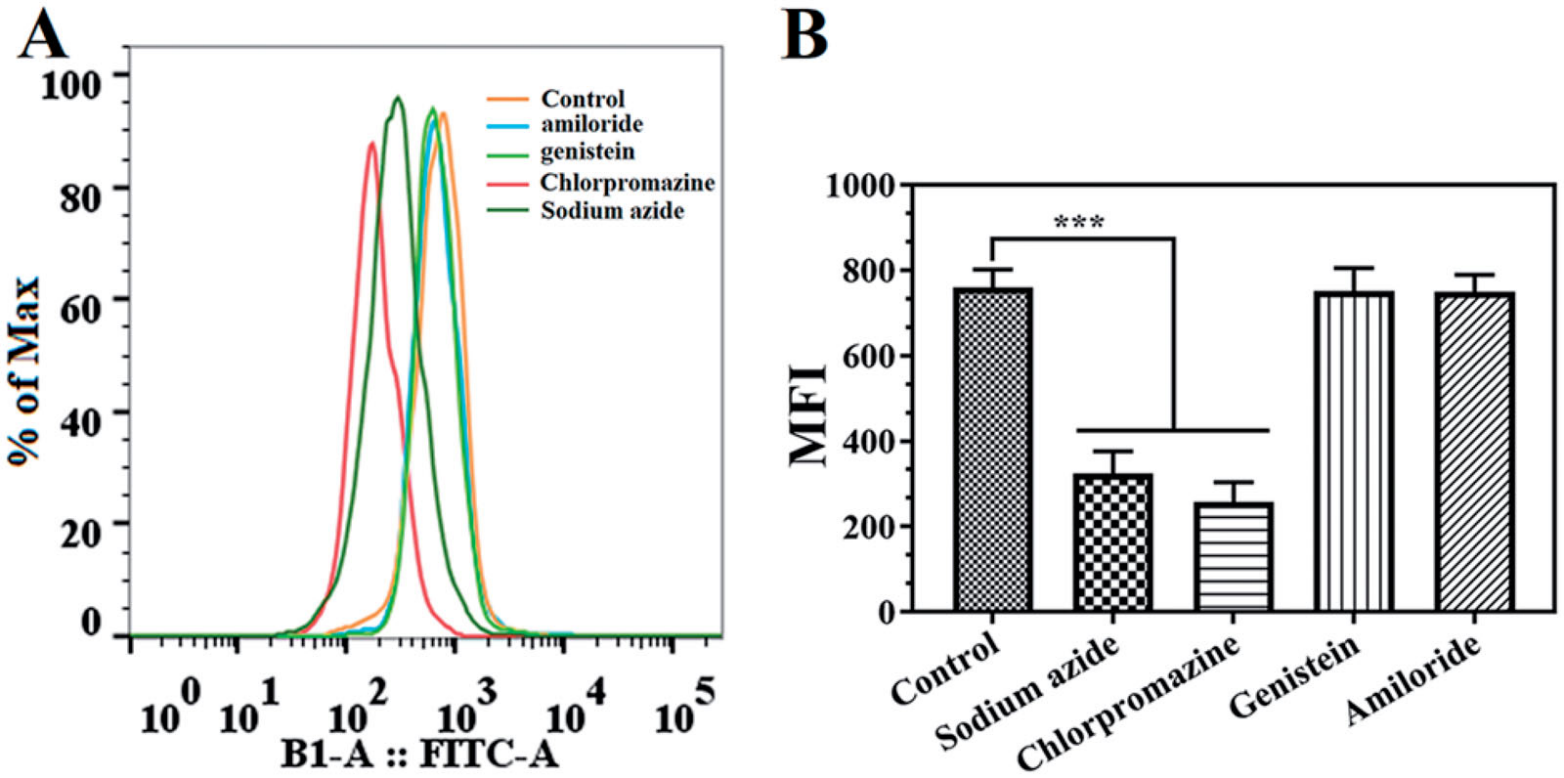
Internalization mechanism of the Cur@HFn. (A) Flow cytometry results with different endocytosis inhibitors and (B) analysis of the average fluorescence intensity (MFI) according to section A, ***p< .001.

In general, cellular uptake efficiency is influenced by several factors, such as different incubation times, different temperatures, different concentrations, and different inhibitors, such as sodium azide (energy-dependent endocytosis), amiloride (micropinocytosis), genistein (caveolae-mediated endocytosis), and chlorpromazine (clathrin-mediated endocytosis). To explore the possible internalization mechanism of Cur@HFn, 4T1 cells were pretreated with various endocytosis inhibitors and the uptake was quantitatively analyzed by flow cytometry. The results are shown in Figure 4(A,B). Compared with the pretreatment group without inhibitors, the cell uptake of Cur@HFn was significantly reduced in the cell group pretreated with sodium azide and genistein (*p* < .001), indicating that the cellular uptake mechanism of Cur@HFn may be related to energy-dependent processes and clathrin-mediated endocytosis, which conforms to the previous reports (He et al., 2020).

### Cell apoptosis and ROS measurement

Generally, apoptosis is one of the main mechanisms leading to the death of cancer cells, making it necessary to evaluate the apoptotic cell potential of the preparation (Singh et al., 2019). The apoptosis of 4T1 cells treated with Cur and Cur@HFn was analyzed using annexin V-FITC/PI flow cytometer. Figure S3A shows the dotted line graph detected by flow cytometry. The lower left quadrant (Q4) corresponds to live cells; the lower right quadrant (Q3) corresponds to early apoptotic cells; the upper right quadrant (Q2) represents late apoptosis, and the upper left quadrant (Q1) contains necrotic cells. The total amount of apoptosis was calculated by Q3 and Q2 (Figure S3B). Compared with the Cur group, Cur@HFn induced a greater proportion of 4T1 cell apoptosis (*p* < .001), reaching 15% of cell apoptosis. The enhanced cancer cell suppression effect of Cur@HFn may be attributed to the high-efficiency targeted cell uptake and cur enrichment to produce excessive ROS (Yu et al., 2021), which is consistent with the above-mentioned *in vitro* cytotoxicity test results. Nuclei were visualized by counterstaining with DAPI (blue, Figure S3C). Compared with the control group, the cells in the drug-treated group showed condensed nuclei and deepened staining. The special morphological (Figure S3D) feature of apoptotic cell was observed with electron microscope, and the volume of cells in the Cur@HFn group was significantly reduced. The morphological results indicated that Cur and Cur@HFn induced apoptosis at an early stage, which was consistent with the flow cytometry results.

**Figure 5.**
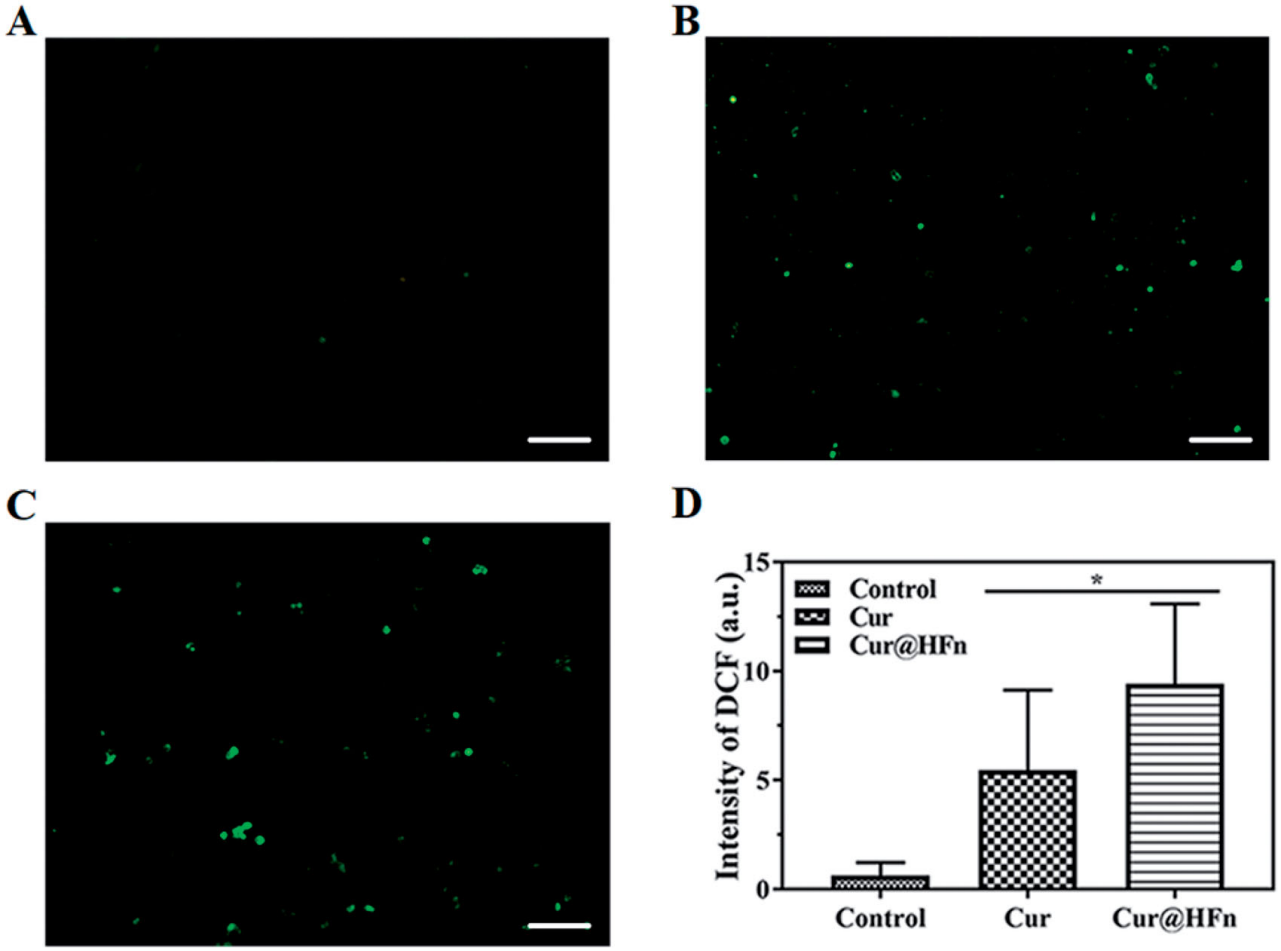
The results of ROS level in 4T1 cells, (A) Untreated control, (B) treated with free Cur, (C) treated with Cur@HFn (scale bar: 100 μm), and (D) quantification of DCF fluorescence intensity has been performed using ImageJ Analysis software (*p< .05 vs. Cur).

Reactive oxygen species are produced by the chemical reduction of O_2_, including superoxide (O_2_·), superoxide anion (O_2_·–), hydroxyl group (·OH), and hydrogen peroxide (H_2_O_2_). ROS are cellular signaling proteins that are involved in every phase of cellular metabolism (Ye et al., 2021b). Cur can increase the production of ROS, leading to many dysfunctions in cells, including DNA damage, apoptosis, and cell cycle arrest in cancer cells (Li et al., 2021). To verify the mechanism of inducing apoptosis, ROS production in cancer cells was detected by a ROS probe. Compared with the free Cur group, the Cur@HFn group produced more green fluorescence in the cells (Figure 5(A–C)). The fluorescence quantitative data (Figure 5(D)) obtained through ImageJ software (Bethesda, MD) showed that the fluorescence intensity between Cur@HFn and free Cur groups was significantly different (*p* < .05). The results showed that Cur@HFn significantly enhanced intracellular ROS in cancer cells, which may be caused by HFn delivering more Cur to the inside of the cell. Therefore, Cur-induced ROS-mediated DNA damage may relate to the induction of cell apoptosis.

### *In vivo* antitumor efficacy study

**Figure 6.**
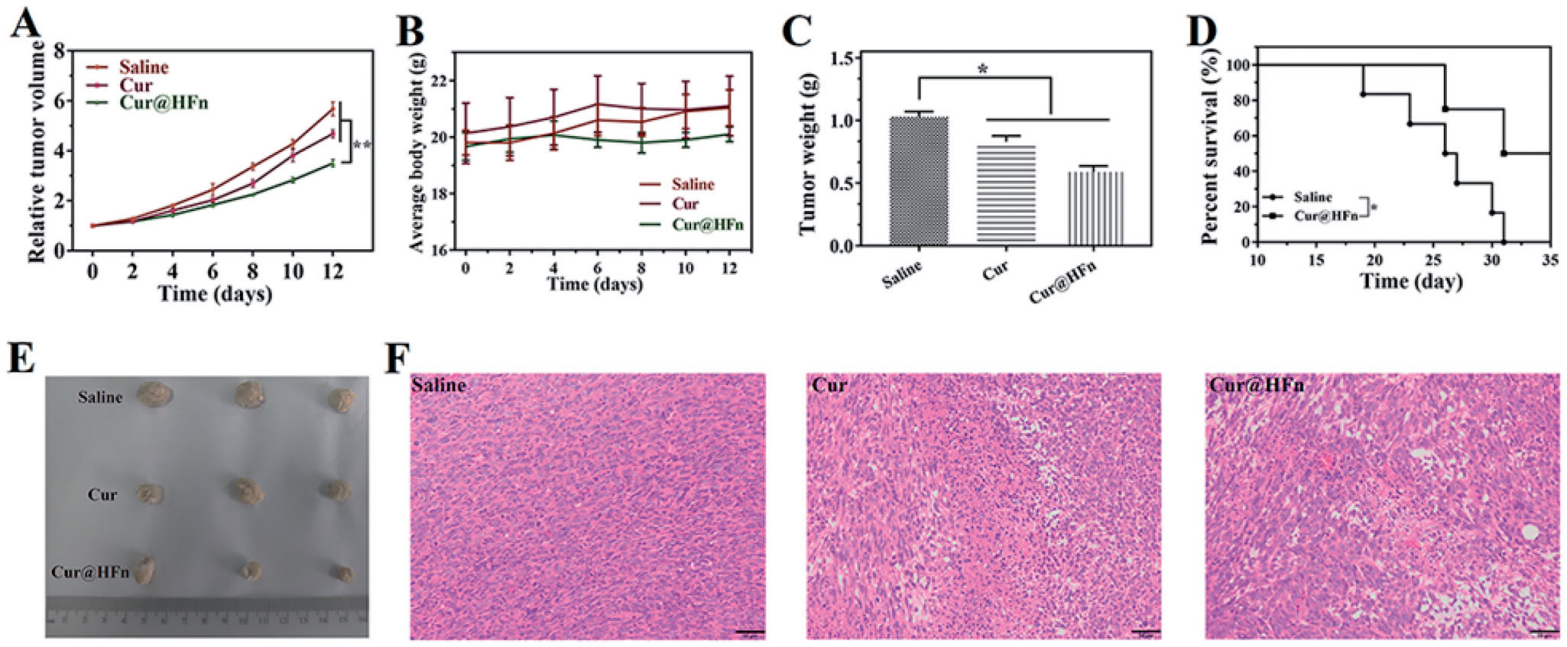
Anti-tumor efficacy of Cur@HFn in 4T1 breast cancer model. (A) Inhibition of tumor growth *in vivo*. (B) Weight change. (C) Weight of isolated tumor after drug treatment. (D) The survival rate of mice with or without treatment. (E) Photographs of tumors obtained after treatments. (F) H&E staining of tumor section at the end of anti-tumor study (scale bar: 50 μm).

Promoted by the inhibitory activity of Cur@HFn and its high cell uptake *in vitro*, 4T1 tumor-bearing mice were used as model animals to further evaluate the anti-tumor activity of Cur@HFn. With normal saline used as a negative control, the tumor volume of each group of tumor-bearing mice in the experiment is shown in Figure 6(A). The results showed that Cur@HFn inhibited tumor growth significantly greater than Cur (*p* < .01). The curve of mouse body weight over time is shown in Figure 6(B). The average weight of mice in each group was relatively stable and slightly increased, indicating that Cur@HFn has no obvious general toxicity. At the end of the experiment, the tumor situation of mice in each group is shown in Figure 6(C,E). The average tumor weight of Cur@HFn is significantly smaller than Cur (*p* < .05). Besides this, the Cur@HFn significantly prolonged the survival rate (Figure 6(D)). To explain the treatment effect from the cellular perspective, H&E staining showed that a large number of cell necrosis with nuclear rupture and nuclear-cytoplasmic atrophy were observed in the tumor tissues of the Cur@HFn treatment group, further indicating that Cur@HFn can effectively inhibit tumor growth (Figure 6(F)). These results indicate that the enhanced anti-tumor effect of Cur@HFn can be attributed to TfR1-mediated tumor-targeted accumulation and pH-responsive continuous Cur release within the tumor, resulting in more necrosis and apoptosis at the cell level (Geninatti Crich et al., 2015). This potential mechanism is in good agreement with the results of cell experiments.

### Safety profiles

**Figure 7.**
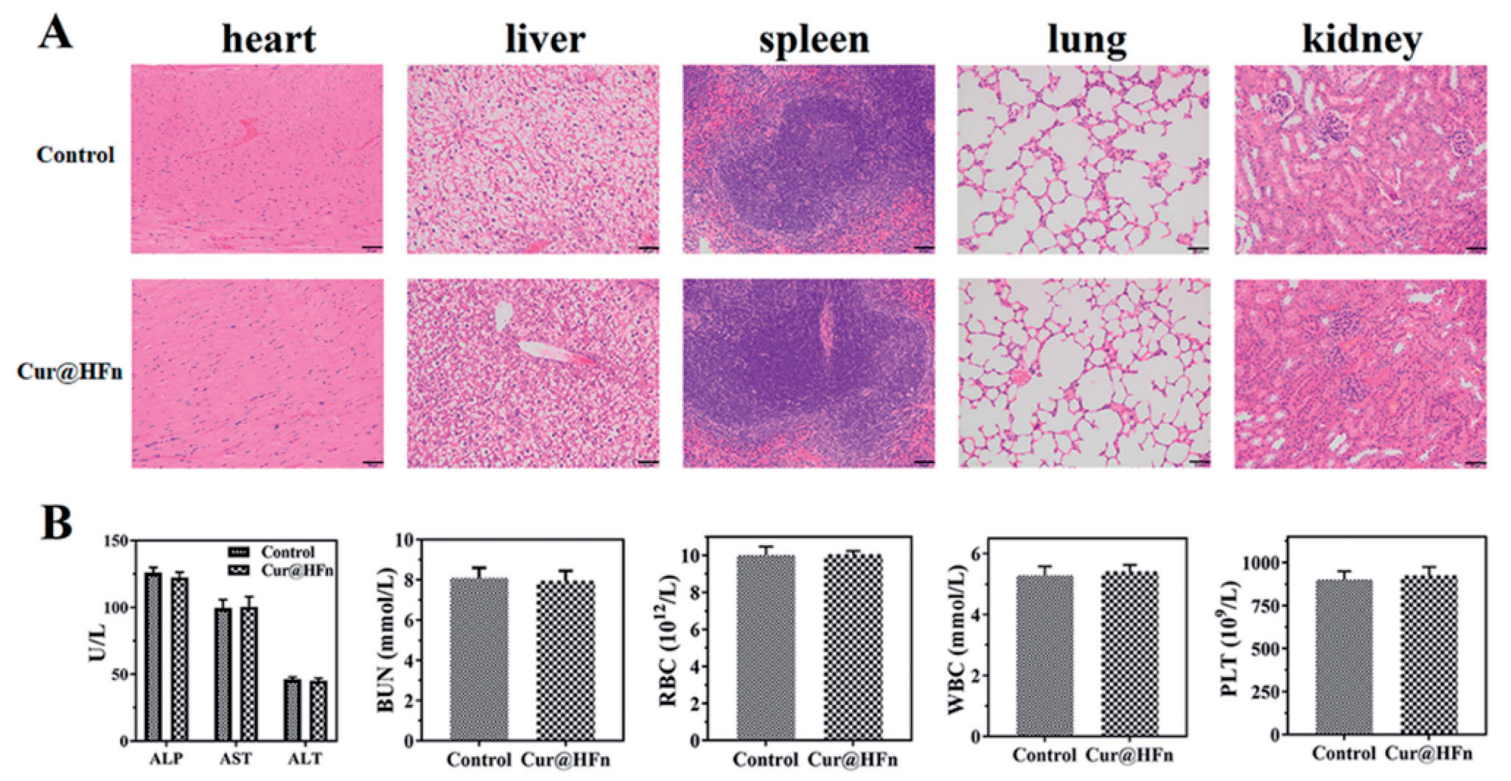
(A) Images of H&E stained major organs tissue sections of saline and Cur@HFn, (B) levels of alanine aminotransferase (ALT), aspartate aminotransferase (AST), alkaline phosphatase (ALP), blood urea nitrogen (BUN), red blood cell (RBC), white blood cell (WBC), and platelets (PLT).

To further assess the toxicity and application potential of Cur@HFn, the tumors in the Cur@HFn treatment group were fixed and H&E stained at the end of the anti-tumor study (Vankayala et al., 2019). The H&E results of the heart, liver, spleen, lung, and kidney (Figure 7(A)) showed that control and Cur@HFn did not cause pathological changes in these organs, and all cells were arranged neatly. After 12 days of treatment in healthy Balb/c mice, blood samples were collected for serum biochemical and complete blood count evaluation. The results (Figure 7(B)) showed that all parameters including ALT, AST, ALP, BUN, RBC, WBC, and PLT were all within the normal range. The above results confirm that Cur@HFn will not damage the liver and kidney function in the long-term treatment of breast cancer, has excellent biocompatibility, and will not cause any adverse reactions.

## Conclusions

It is favorable that Cur, a traditional Chinese medicine, has anti-cancer effects, especially for breast cancer. However, Cur has poor water solubility, photodegradation, serious side effects, low cellular uptake, and a lack of targeting selectivity, which limits its application in clinical anticancer applications. HFn does not require any further functionalization and can be used as an effective carrier for treatment for various medical purposes. Therefore, this study constructed a targeted breast cancer drug delivery platform based on HFn nanocage to improve the anti-cancer performance of Cur. Cur@HFn exhibits high stability and pH-responsive drug release behavior, and the release amount under acidic conditions is much higher than that under neutral conditions. Cur@HFn showed stronger cytotoxicity and cellular uptake in breast cancer cell models and improved internalization efficiency through clathrin-mediated endocytosis. In addition, *in vivo* anti-tumor experiments in 4T1 tumor-bearing mice showed that Cur@HFn has lower systemic toxicity and higher *in vivo* therapeutic effects. As a consequence, our findings suggest that Cur@HFn can become a low-toxicity and high-efficiency nano-DDS for the treatment of breast cancer.

## Supplementary Material

Supplemental Material
