# Supplementary material for: Selective delivery of curcumin to breast cancer cells by self-targeting apoferritin nanocages with pH-responsive and low toxicity
Source: Drug Deliv. 2022 Apr 1;29(1):986–96. doi: 10.1080/10717544.2022.2056662 (PMC8979518; doi:10.1080/10717544.2022.2056662)
Supplement: Supplemental Material [file IDRD_A_2056662_SM9230.docx]

**SUPPORTING INFORMATION FOR THE MANUSCRIPT**

**Selective delivery of curcumin to breast cancer cells by self-targeting apoferritin nanocages with pH-responsive and low toxicity**

Peng Ji ^a^, Xianglong Wang ^a^, Jiabing Yin ^a^, Yi Mou ^a^, Haiqin Huang ^b*^, Zhenkun Ren ^c*^

(^a^College of Pharmacy and Chemistry & Chemical Engineering, Jiangsu Provincial Key Laboratory of Chiral Pharmaceutical Chemicals Biologically Manufacturing, Taizhou University, Taizhou 225300, PR China; ^b^School of pharmacy, Nantong University, Nantong 226000, PR China; ^c^The Third Hospital Affiliated of Jinzhou Medical University, Jinzhou 121000, PR China)

**1. Cytotoxicity of nanocarrier HFn**


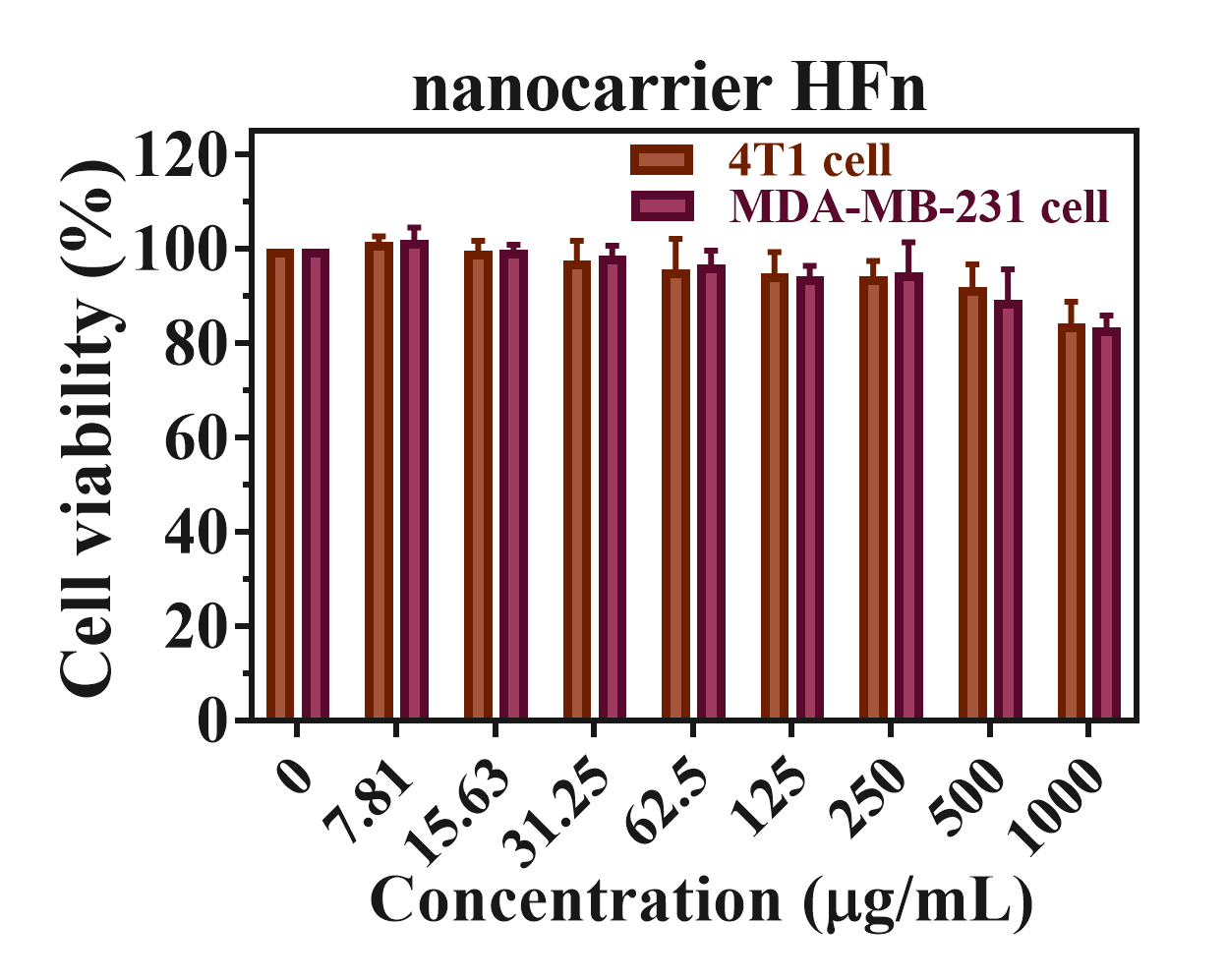


**Figure S1** *In vitro* cytotoxicity evaluation of nanocarrier HFn in MDA-MB-231 and 4T1 cells (Mean ± SD, n= 3).

**2. Fluorescence microscopy images of 4T1 cells after being incubated with Cur and Cur@HFn**


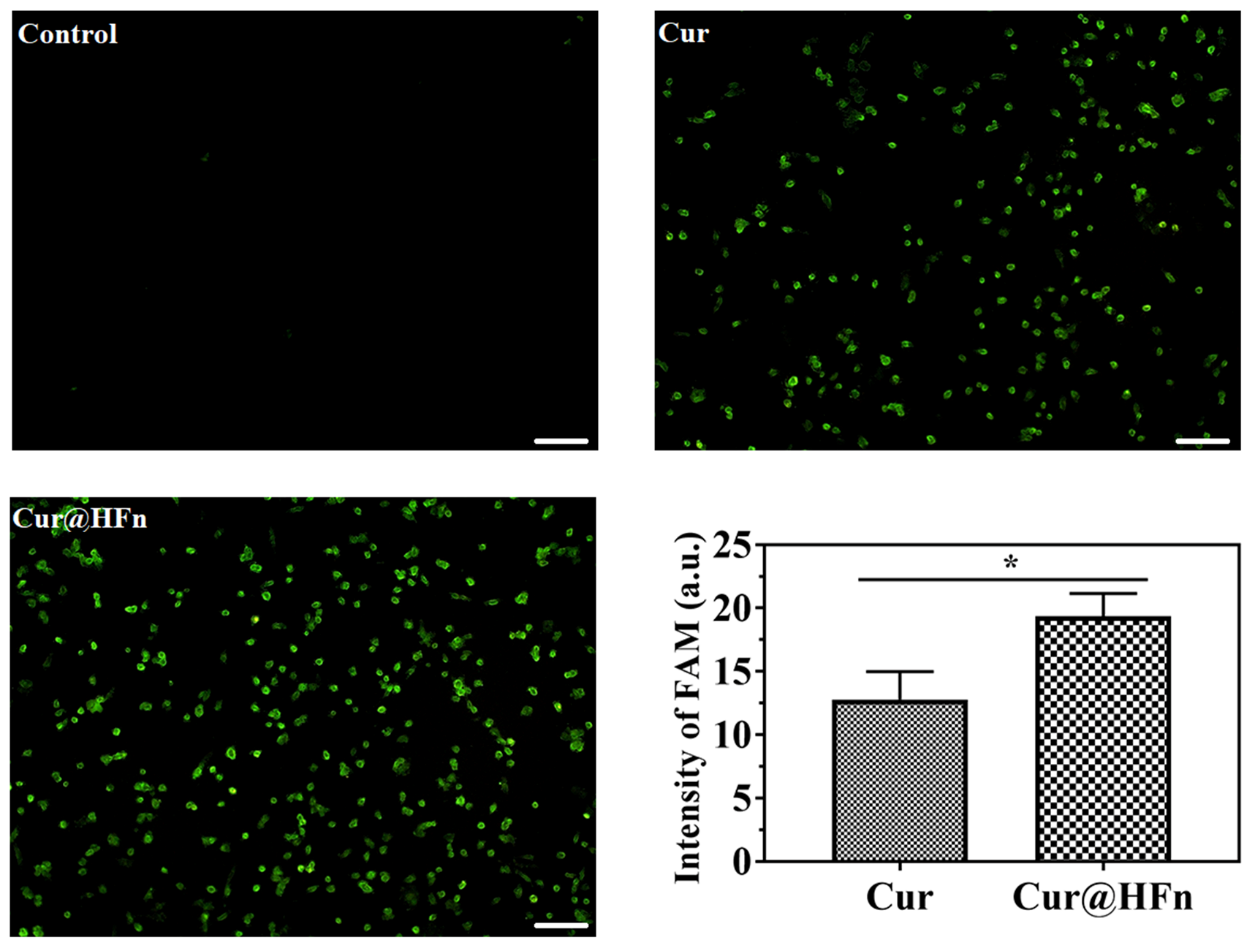


**Figure S2** Fluorescence microscopy images of 4T1 cells after being incubated with Cur and Cur@HFn for 6 h (scale bar: 100 μm). The mean fluorescence intensity of Cur and Cur@HFn, **p*< 0.05.

**3. Cell Apoptosis**


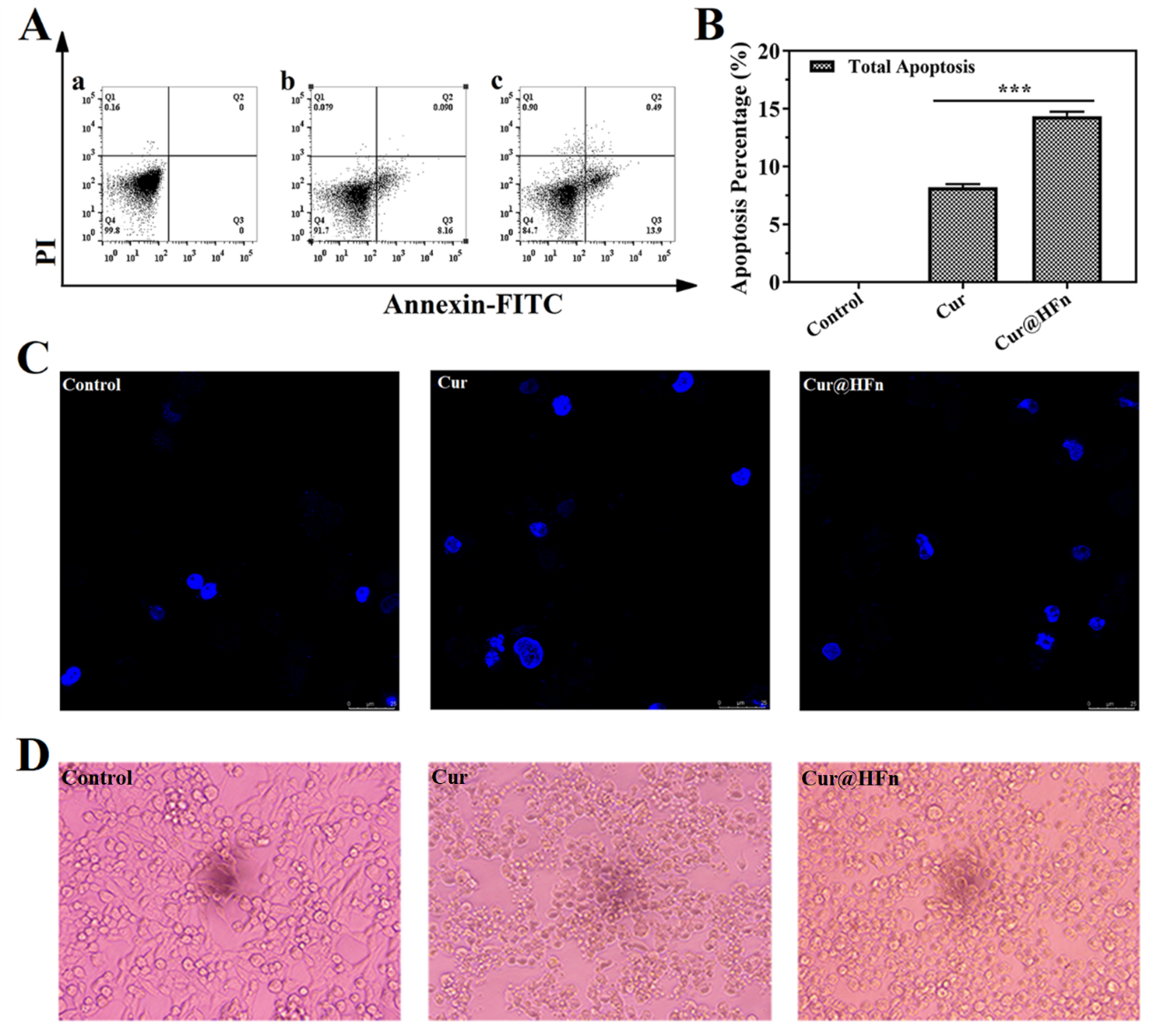


**Figure S3** (A) Flow cytometry analysis of 4T1 cell apoptosis induced by (a) Control, (b) Cur, and (c) Cur@HFn using Annexin V-FITC/PI staining. (B) Quantitative analysis of the total population of apoptotic cells for each group according to A, ****p*< 0.001. (C) The morphological of apoptosis by taining. (D) The special morphological feature of apoptotic cell was observed with electron microscope.
